# Indents

**Published:** 1919-01

**Authors:** 


### INDENTS

**☞Brief Articles of Technical or Scientific Value will receive notice and comment. Contributions invited.**

---

THE TOOTHFORM AREA. A complete analysis of a face must be made to create a harmony of line, even though this harmony be later distorted in the adjustment of the mouth, if we are confronted with deformity or physical limitations which may or may not be disfiguring, or again a condition which is often more difficult because there is no deformity at all.

These variations from the harmonious and symmetrical are what may be designated as "harmonious discords," and can only be recognized by the establishment of a bilateral symmetry accomplished by the comparison of the two halves of the face between certain established lines. Therefore, that portion of the face which is most characteristic, and at the same time nearest to the teeth themselves, should be selected, because this area is less affected by high lights, shadow, or style of wearing the hair, etc. The discordant element which enters into the question must be applied after harmony has been recognized.

For this reason the area I have established for my convenience, is from the eyebrows to the commisure of the mouth with the median line vertically through the nose at right angles with the horizontal lines, which are parallel to each other.

It should be borne in mind that what we often select as an example of harmonious discord, or an individuality is but the result of a digression from the symmetrical and ideal, and the effect that such a condition must necessarily have on facial expression. Therefore, if possible, it is our function to restore this unusual or characteristic individuality, and like all reproductions of nature, it is an art of the highest type.—Dr. Essig, *Journal N. D. A.*

---

Devitalizing Pulps. I practically never devitalize a pulp unless it is exposed and diseased. I use either cocaine or novocaine by the pressure method or by injection. I formerly devitalized a great many teeth by pressure anesthesia only. The best way to do this is to drill through the enamel with a small stone to sound dentine, at such a place as will give access to the dentinal canals most directly. Then drill a pit into the dentine a short distance with a small bud shaped bur. Moisten this pit with a saturated solution of cocaine with adenalin and force the solution into the dentine with a small plug of unvulcanized rubber by a suitable plugging instrument. A piece of gutta percha stuck to the end of an amalgam instrument will do. Hold the pressure for a short time, then drill the pit as much deeper as can be done without pain. Repeat the pressure anesthesia as before, until the pulp is reached and uncovered without sensation. The process, if carried by distinct steps will serve to desensitize dentine as well as for exposing and completely anesthetizing the pulp with little danger of incurring infection.—Dr. Smedley in *Dental Digest.*

---

The Use of Natural Teeth for Crowns. Natural teeth have not been used in the mouth on account of the loss of color and their liability to caries.

I believe I have solved these difficulties. All that is necessary is to saw through a natural tooth preserved aseptically in formalin in the region of the neck; then the contents of the crown are cleared out with a bur; the dentine having been entirely removed, the colorless shell of enamel remains. Having made certain that there is no fissure in the enamel this shell is adjusted on the model. The color having been determined it only remains to fill the tooth with a cement of the desired shade. The synthetic cements give good results, and the appearance is very likelife.

The teeth thus prepared are fixed to the root with a band so as to avoid salivary infiltration. In this way the color difficulty is solved.

The caries difficulty is solved because it will remain localized to the enamel and can always be repaired by fillings.—M. Hulin in *L'Odontologie,* Aug., 1918.

---

Are You in a Profession or Are You Following an Occupation? If dentistry is a profession, it ought to be characterized by professional training, professional ideals, professional honor, and professional pride. I know that in this city and in every city of any size there are people who are following the occupation of dentist without the slightest regard for those things of which every honorable man who has the title of Doctor of Dental Surgery ought to be proud, and I tell you, gentlemen, that these things that you know of and I know of are going on in the city of Chicago and in the state of Illinois right now to the detriment of your profession and to the disgrace of the honest name of dentistry, with reflection on you and on every other man or woman called a dentist.

Now, can we stop advertising dentists? I tell you this old world of ours is undergoing a great change Two years ago the American people were carefree and indifferent. We have changed our mode of thought, the American people. I was chairman of a republican committee down in Ohio when the republican party in that state was dominated by a saloonkeeper of Cincinnati and there was not a single person in the republican party who dared to get up and say, "You shall not lead us." But within two years, within a few months, you have seen a change. Today there is no one so poor as to bow before a saloonkeeper. Our nation has determined to bar intoxicants. This change was found necessary for the period of the war, in order that American manhood might have the efficiency to fight the foe, and there is no one who raised one single voice against it. Why can we not stop newspaper advertising? There are two ideals in the American mind today. One is "efficiency" and the other "fitness." Is he fit to fight for the flag of his country? Oh! the shame and disgrace that comes to an American citizen when he is told, "You are unfit, unfit to fight for the life of the country that gives you a home, that gives you shelter, that gives you an opportunity to live." Are you fit? I tell you that we can go before the people of Illinois now and demand things we could not do two years ago. When this war is over there is going to be a different citizenship and a different aspect towards life, and a different viewpoint on many questions. You know how your own thought is reshaping and changing and climbing upward as our boys are fighting on the western front. Now is the time to strike. Come with us. If you will help the Department of Registration and Education we will show the people of Illinois whether we cannot stop some of those infernal outrages that have been perpetrated in this city and this state under the name of an honorable profession.—Dr. Shepardson in *Dental Review.*

NERVOUS SHOCK IN DENTISTRY. It would appear that there is nothing today that the dentist should give more attention to than the prevention of shock in all cases, even down to the prevention of shock to the dental pulp through the placing of metallic fillings too closely approximating the pulp. The day of long, tedious dental operations is passing away as civilization advances, banishing those things detrimental to the human economy; so must we adopt methods that will make man's stay upon this sphere more endurable, for as life becomes more complex and the pace gets harder for the human to make, his resistance to hardship and pain becomes less. "The species of animal now dominating the world more completely than any other is at this moment in a state of abnormal activity. The backwash of the European war is seen in the quickened pulse everywhere, even in this land. Not only in war, but in peace also, the complicated machinery of civilization created by man often drives him to his own destruction." Fortunately, science is advancing apace, and holds out means to us with which to combat this tendency of the race—that is, his lessened resistance to pain, and along with it, shock. Especially is this true in reference to anesthesia, which is on a more standardized basis than ever before, thanks to the marvellously-advanced methods that are being developed for us, if we will only avail ourselves of them. I am sorry to say that the cost price seems to be the gulf that exists between ourselves, the patient and their more general use.

Among the cases which come under the dental surgeon's care, in which shock may likely occur, we may class the following:

*First.* The lancing of abscesses, especially deep-seated ones, of which we are called upon to treat quite a few. I am led to place this condition first as a shock producer, as I remember in my own experience, I believe, that this condition causes more shock, fainting and anemia to patients than any other, as by the time the abscess has pointed, the patient has become so debilitated and exhausted, and resistance is so lessened that he readily succumbs to the effects of shock.

*Second.* I would place extraction operations, both ordinary and long, tedious ones. Even in simple extractions, patients often have every symptom of shock before ever the operation is started; this is a case of gaining the patient's confidence to relieve the condition, by not too showy use of instruments, and the hastening of the operation.

*Third.* In cases where we have the misfortune to break off a tooth in extraction, and spend minute after minute working to get the root, driving the patient nearer and nearer to the shock stage all the while. These cases are the ones that should be treated with an anesthetic, especially a general one.

*Fourth.* Exposure of pulp sometimes causes a great deal of shock, sometimes by merely a touch, so that it is advisable to use a short anesthetic and avoid this condition.

*Fifth.* In the case of bed-ridden patients, shock is to be looked for, and as seriously avoided as the given conditions will permit, remembering that their lessened resistance makes them more susceptible.

*Sixth.* Prolonged dental operations, which it should hardly be necessary to mention, as this feature of dentistry is, or should be, already understood by dentists.

Most of us have had cases of shock in our practices, although in most instances the condition is usually emotional or psychical and is easily handled, though this is not always the case. I might cite several cases that I have met within my own practice. They are mostly simple conditions, and I might say that I never have had a case of severe shock.—R. L. Wheeless in *Summary*.

---

Clasps for Partial Dentures. In view of the fact that as root work is not only an expensive procedure, but the number of roots which can be filled ideally are limited, we must have recourse to some satisfactory method of replacing lost organs of mastication, that those who are not in a position to spend large sums on removable bridges may also be served satisfactorily.

This can be done by means of the partial plate of metal or rubber, together with an oscillating clasp which adjusts itself automatically, and brings no stress upon the teeth which serve for the purpose of stability.

I have devised quite an assortment of varied forms of clasps, having a reciprocating or compensating form of movement, and these I find are a wonderful source of comfort to the patient,who is rarely conscious of this form of attachment, even when worn for the first time.—N. S. Essig in *Journal N. D. A.*

---

ANTICIPANT DENTURES. I have been in the habit of making my temporary denture before the teeth are extracted, and have frequently had my patients come directly to my office from the extractor, to have the denture adjusted, if, indeed, adjustment was necessary.

After the shrinkage has taken place to the extent that the temporary piece becomes loose, I have but to use the plate as an impression cup, and run a bite over the teeth to insure their accurate replacement, and remake the plate. This is simple, easy, and very quickly done.—Dr. Essig in *Journal N. D. A.*

